# Genome-wide identification, transcriptional profiling, and miRNA-binding site analysis of the *LBD* gene family in the camphor tree

**DOI:** 10.3389/fpls.2025.1591736

**Published:** 2025-06-19

**Authors:** Jiaqi Zhang, Luyang Shan, Haoran Qi, Caihui Chen, Yongda Zhong, Meng Xu

**Affiliations:** ^1^ State Key Laboratory of Tree Genetics and Breeding, Co-Innovation Center for Sustainable Forestry in Southern China, Nanjing Forestry University, Nanjing, China; ^2^ Jiangsu Key Laboratory for the Research and Utilization of Plant Resources, Institute of Botany, Jiangsu Province and Chinese Academy of Sciences (Nanjing Botanical Garden Mem. Sun Yat-Sen), Nanjing, China; ^3^ Jiangxi Provincial Key Laboratory of Improved Variety Breeding and Efficient Utilization of Native Tree Species, Institute of Biological Resources, Jiangxi Academy of Sciences, Nanchang, China

**Keywords:** camphor tree, Lateral Organ Boundaries (LOB), microRNA (miRNA), transcription factors, miRNA targeting

## Abstract

*Cinnamomum camphora* (Lauraceae), an evergreen arborescent species endemic to East Asian ecosystems, is ecologically and economically prized for three cardinal attributes: decay-resistant xylem, aesthetic canopy architecture, and pharmacologically active terpenoid emissions. The plant-specific Lateral Organ Boundaries Domain (LBD) transcription factors mediate phylogenetically conserved developmental pathways governing lateral organogenesis and secondary metabolism across embryophytes. Despite multiple published *C. camphora* genome assemblies, functional characterization of LBD transcription factors in this species remains limited. We systematically identified 40 *LBD* genes through whole-genome analysis and characterized their structural features, evolutionary relationships, and expression patterns. Five are intron-free, while seven genes harbor two or more introns each. Detailed annotation of *CcLBD* promoter regions identified 33 cis-regulatory elements linked to hormone signaling and stress adaptation. Transcriptional dynamics of the 40 *CcLBD* genes were profiled across seven tissues of the camphor tree using short-read RNA-Seq, revealing that 22 genes were highly expressed in flowers and 12 were predominantly expressed in roots, suggesting potential roles in reproductive organ development and root formation in *C. camphora*. Phylogenetic analysis classified all CcLBD proteins into two clades, each harboring a conserved lateral organ boundaries (LOB) domain. Integrative omics analyses (small RNA-seq and degradome data) further implicated miR408 and miR2950c in post-transcriptional regulation of *CcLBD5* via mRNA cleavage. These results establish a framework for the functional dissection of LBD-mediated developmental and stress-response pathways in *C. camphora*.

## 1 Introduction

Lateral organ boundaries domain (LBD) proteins, alternatively termed ASYMMETRIC LEAVES2-LIKE (ASL) proteins, are defined by a conserved lateral organ boundaries (LOB) domain (Iwakawa et al., 2002). This structural module contains three conserved elements: a zinc finger-like CX2CX6CX3C motif essential for DNA binding, a Gly-Ala-Ser (GAS) block, and a leucine zipper motif LX6LX3LX6L critical for protein dimerization (Majer and Hochholdinger, 2011). Phylogenetic analyses classify the LBD family into Class I (retaining functional leucine zippers) and Class II (lacking this motif), with Class I subdivided into five evolutionarily distinct subclasses (Ia-Ie) that constitute the majority of LBD members (Landschulz et al., 1988; Iwakawa et al., 2002; Shuai et al., 2002; Chanderbali et al., 2015; Liu et al., 2019).

As plant-exclusive transcription factors, these proteins orchestrate lateral organ formation in shoots and roots. They also regulate biological processes, including tissue regeneration and pollen maturation (Oh et al., 2010; Majer and Hochholdinger, 2011; Fan et al., 2012; Kim et al., 2015; Xu et al., 2016). AtLBD18 interacts with auxin response factors AtARF7 and AtARF19, enhancing their transcriptional activity to promote lateral root development (Pandey et al., 2018). Ectopic expression of *AtLBD30 *and *AtLBD18 *drives nonvascular cell reprogramming into tracheary elements, the core structural components of xylem vessels (Soyano et al., 2008). Recently, a phylogenetically related LBD member, similar to *Arabidopsis* AtLBD17 and AtLBD29, has been identified as crucial for lateral root and adventitious root development in tomato (*Solanum lycopersicum*) (Omary et al., 2022). Furthermore, another member of the Class IB LBD transcription factors, MtLBD16 in *Medicago truncatula* and ASL18/LjLBD16a in *Lotus japonicus*, plays critical roles in lateral root development and nodule formation (Schiessl et al., 2019; Soyano et al., 2019). Additionally, LBD and NAC proteins exhibit a crucial positive feedback regulatory mechanism essential for the growth modulation of *Arabidopsis thaliana* by regulating xylem cell differentiation (Soyano et al., 2008; Yamaguchi et al., 2010). In woody plants, LBD transcription factors are pivotal for secondary vascular development. Overexpression of *PtaLBD1* in *Populus tremula × P. alba* promotes secondary phloem and xylem growth (Yordanov et al., 2010). In *P. trichocarpa*, PtrLBD39 and PtrLBD22 contribute to tension wood formation by mediating transcriptional responses to mechanical stress (Yu et al., 2022), while in *Eucalyptus grandis*, EgLBD22, EgLBD29, and EgLBD37 regulate phloem and xylem differentiation, influencing wood formation (Lu et al., 2018). Despite these advances, the functional characterization of LBD transcription factors in perennial woody species remains limited. Given the unique developmental traits of trees, including sustained secondary growth, vascular complexity, and long-term environmental adaptation, further research is needed to elucidate the evolutionary and functional diversification of LBD transcription factors in woody lineages.

MicroRNAs (miRNAs) are evolutionarily conserved small non-coding RNAs (18–24 nucleotides) that fine-tune gene expression post-transcriptionally (O’Brien et al., 2018). By binding complementary mRNA sequences, they induce transcript cleavage or translational repression, thereby modulating diverse biological processes (Rong et al., 2024). In *Medicago truncatula*, the miR390/TAS3 module regulates MtLBD17/29a via ARF2, coordinating lateral root and nodule development (Kirolinko et al., 2024). Similarly, in poplar, miR408 suppresses *LBD15* expression, influencing lignification and biomass accumulation (Guo et al., 2023). While several miRNAs indirectly regulate *LBD* genes by targeting upstream transcription regulators (e.g., NAC1 or ARF family members), no direct miRNA–LBD interactions have been experimentally validated.

The Lauraceae family includes ecologically and economically vital species that dominate tropical and subtropical forests, serving as key resources in timber, medicinal compounds, spices, and essential oils. *Cinnamomum camphora* (camphor tree) is a particularly valuable species due to its aromatic properties and ecological significance (Meng et al., 2021). Widely cultivated in East Asia, it is a cornerstone of subtropical evergreen broadleaved forests and a major global producer of essential oils (Wang et al., 2022).

Despite the availability of multiple high-quality *C. camphora* genome assemblies (Wei-Hong et al., 2021; Jiang et al., 2022; Wang et al., 2022; Li et al., 2023), the functional roles of its LBD transcription factors remain largely unexplored. In this study, we present the first genome-wide identification and characterization of 40 *LBD* genes in *C. camphora*. Our comprehensive analysis revealed conserved LOB domain architectures, tissue-specific expression divergence, and cis-regulatory element landscapes within promoter regions. Evolutionary assessments resolved phylogenetic relationships among LBD subfamilies, while integrated sRNA-degradome data uncovered post-transcriptional regulation of *CcLBD5* mediated by miR408/miR2950c through cleavage events. These findings provide a foundational framework for investigating *LBD* gene function in woody plants and shed new light on multilayered regulatory networks to govern organ development and stress responses in perennial species.

## 2 Materials and methods

### 2.1 Plant materials

The tissues and organs utilized in this study were sourced from there-year-old seedlings propagated from cultivar ‘Gantong1’ cuttings cultivated at the Jiangxi Academy of Sciences Nursery Base. To ensure reliability and statistical significance, three biological replicates were performed.

### 2.2 Sequence alignment and homolog identification

The genome and protein sequences of *C. camphora* cultivar ‘Gantong1’ were sourced from our prior study (https://ngdc.cncb.ac.cn/gwh/Assembly/23429/show) (Shen et al., 2022), which were then employed to construct a local BLAST database. Homology screening employed *A. thaliana* LBD proteins from TAIR as BLASTp queries, with a stringent e-value threshold of 1e^-20^ (Lamesch et al., 2011). Candidate LBD proteins in ‘Gantong1’ were screened using the LOB domain-specific hidden Markov model (PF03195) (Mistry et al., 2020). Domain validation was performed via SMART and HMMER tools under default settings (Finn et al., 2011; Letunic et al., 2011).

For functional characterization, the Expasy Protparam server predicted coding sequences (CDS), isoelectric points (pI), molecular weights (MW), and hydropathy indices (GRAVY) of all CcLBDs (Kyte and Doolittle, 1982; Wilkins et al., 1999). Subcellular localization predictions were further conducted using Cell-PLoc 2.0 (Chou and Shen, 2008).

### 2.3 Phylogenetic and conserved domains analysis

Protein sequences of *A. thaliana* and *C. camphora* were aligned with ClustalX 2.0 (Larkin et al., 2007). A Neighbor-Joining phylogenetic tree was constructed in MEGA-X, with node support evaluated by 1000 bootstrap replicates (Saitou and Nei, 1987; Kumar et al., 2018). Conserved motifs were identified through MEME suite analysis, configured to detect motifs of 6–50 amino acid widths with a six-motif limit per sequence (Bailey et al., 2009). Sequence logos representing motif conservation patterns were generated via WebLogo (Crooks et al., 2004).

### 2.4 Gene structure and promoter Cis-acting elements

The chromosomal distribution of *CcLBD* genes was analyzed using TBtools (v1.1047) (Chen et al., 2020). Gene structures (exon/intron organization) were annotated with GSDS 2.0, followed by visualization in TBtools (Hu et al., 2014; Chen et al., 2020). Promoter sequences (2000 bp upstream) were extracted from the *C. camphora* genome via Tbtools (Chen et al., 2020). Cis-regulatory elements were predicted using PlantCare, with statistically filtered results presented as a heatmap (Lescot et al., 2002).

### 2.5 Gene expression analysis

Transcriptome sequencing data from multiple *C. camphora* tissues (flowers, leaves, fruits, roots, young stems, developing xylem, and trunk phloem) were obtained from our previous study (Shen et al., 2022). Expression levels were normalized and converted to log_10_(TPM+1). Candidate *CcLBD* expression profiles were analyzed using RNA-Seq short-read data and visualized through pheatmap (v1.0.12).

For experimental validation, total RNA was isolated from high-polyphenol/polysaccharide-enriched tissues with the RNAprep Pure Plant Plus Kit (Tiangen), followed by first-strand cDNA synthesis (TaKaRa 5X PrimeScript™ RT Master Mix). Gene-specific primers designed via Beacon Designer 8 enabled qRT-PCR amplification (ViiA 7 Real-Time PCR System, Applied Biosystems) using PowerUp™ SYBR™ Green Master Mix. Relative expression levels were normalized to reference genes via the 2^−ΔΔCt^ method.

### 2.6 Prediction and identification of miRNA-binding sites

Degradome and small RNA sequencing data (Accession: SRP127892) (Chen et al., 2018) were analyzed to identify miRNA-*CcLBD* interactions. Potential miRNA binding sites were predicted using psRNATarget (https://www.zhaolab.org/psRNATarget/). High-confidence targets were defined as alignments with CleaveLand4 (v4.5) (Addo-Quaye et al., 2008) category ≤2 and p-value ≤0.05.

## 3 Results

### 3.1 Identification of *LBD* genes in *C. camphora*

Through a systematic genome-wide investigation of *C. camphora*, we identified 40 unique *LBD* genes, designated ​*CcLBD1*​ to ​*CcLBD40*​ based on their chromosomal distribution. The encoded proteins exhibited substantial size diversity, with molecular weights spanning ​12.53 kDa​ (CcLBD4, 109 amino acid residues) to ​45.06 kDa​ (CcLBD3, 398 amino acid residues). Theoretical pI values spanned from 4.71 (CcLBD20) to 9.69 (CcLBD15), highlighting substantial electrostatic divergence within the LBD family. A striking 95% of family members exhibited negative GRAVY indices (-0.89 to -0.12), consistent with hydrophilic character. Subcellular localization predicted by Cell-PLoc uniformly placed all LBD members within the nuclear compartment (**Table 1**).

**Table 1:** Details of the 40 *CcLBD* genes and their encoded proteins in *C. camphora*.

Gene Name	Type	Chromosome location	Size (aa)	MW (kDa)	pI	Stability	A.I.	GRAVY
*CcLBD1*	la	Chr1:8359979-8361791	175	19.33	8.17	U	83.09	-0.179
*CcLBD2*	ld	Chr1:14993042-15001269	269	30.46	5.67	U	68.18	-0.666
*CcLBD3*	ld	Chr1:15723412-15730997	398	45.06	8.83	U	72.81	-0.563
*CcLBD4*	ld	Chr1:15736205-15736534	109	12.53	8.27	U	89.54	-0.313
*CcLBD5*	lla	Chr1:19067525-19068666	206	22.34	6.73	U	82.82	-0.038
*CcLBD6*	ld	Chr1:36039397-36042231	320	36.29	8.65	U	64.72	-0.707
*CcLBD7*	ld	Chr1:53330647-53332656	243	26.98	8.98	U	67.86	-0.626
*CcLBD8*	ld	Chr1:53850079-53851094	290	32.64	6.02	U	64.28	-0.717
*CcLBD9*	lb	Chr2:57226937-57227743	268	29.62	6.42	U	64.89	-0.567
*CcLBD10*	lla	Chr2:68870803-68872010	171	18.5	6.12	U	87.84	0.077
*CcLBD11*	la	Chr3:2330405-2332026	168	19.14	6.65	U	69.82	-0.374
*CcLBD12*	lb	Chr3:8071011-8071502	163	18.11	7.59	U	66.44	-0.338
*CcLBD13*	la	Chr3:8139220-8140194	163	18.17	6.8	U	84.97	-0.273
*CcLBD14*	lb	Chr3:9725267-9727969	214	23.28	9.01	U	70.7	-0.379
*CcLBD15*	lc	Chr3:63919804-63926126	293	32.36	9.69	U	75.9	-0.328
*CcLBD16*	la	Chr3:73912118-73913141	151	16.79	5.6	U	81.99	-0.134
*CcLBD17*	lb	Chr3:76539283-76545469	198	21.57	8.56	U	72.02	-0.294
*CcLBD18*	la	Chr4:659563-660417	186	21.82	5.13	U	63.98	-0.558
*CcLBD19*	lb	Chr4:8434799-8435356	185	20.51	7.63	U	82.27	-0.18
*CcLBD20*	la	Chr4:41674877-41677037	150	17.25	4.71	U	65.07	-0.599
*CcLBD21*	lla	Chr4:42947289-42950023	202	21.18	8.09	U	80.25	-0.006
*CcLBD22*	llb	Chr4:54869036-54870478	258	28.13	6.75	U	80.23	-0.428
*CcLBD23*	la	Chr4:59494503-59498005	170	18.58	8.84	U	67.71	-0.246
*CcLBD24*	la	Chr4:61649542-61650757	195	21.16	6.93	U	82.67	-0.085
*CcLBD25*	lc	Chr5:46480564-46483135	302	33.02	6.24	U	79.77	-0.362
*CcLBD26*	la	Chr5:52449637-52454301	170	18.75	8.31	U	71.18	-0.216
*CcLBD27*	la	Chr5:55318452-55319478	200	21.47	6.81	U	76.2	-0.228
*CcLBD28*	lc	Chr6:9543841-9546388	228	24.43	8.26	U	83.03	-0.149
*CcLBD29*	lc	Chr7:7695554-7696545	172	18.55	8.68	U	89.07	0.008
*CcLBD30*	lb	Chr7:17769019-17769831	270	30.2	6.51	U	62.22	-0.659
*CcLBD31*	la	Chr8:45228864-45230358	153	17.47	6.63	U	59.41	-0.424
*CcLBD32*	lb	Chr9:19415435-19417005	213	23.21	8.84	U	79.25	-0.299
*CcLBD33*	lc	Chr9:38676439-38679498	200	21.79	6.93	U	80.1	-0.105
*CcLBD34*	lc	Chr9:38713962-38714878	234	25.68	5.8	U	67.22	-0.255
*CcLBD35*	llb	Chr10:38816567-38818478	300	32.51	6.66	U	75.5	-0.371
*CcLBD36*	la	Chr11:7560409-7561961	212	23.19	5.32	U	71.32	-0.265
*CcLBD37*	lc	Chr11:10153359-10154258	219	24.27	5.35	S	78.9	-0.177
*CcLBD38*	llb	Chr11:30541082-30542701	302	32.9	6.2	U	81.36	-0.298
*CcLBD39*	la	Chr12:31608996-31609956	188	21.34	5.79	U	81.49	-0.417
*CcLBD40*	ld	Chr12:31986997-31987827	196	22.25	5.76	U	62.3	-0.646

MW, molecular weight; pl, isoelectric point; A.I., aliphatic index; GRAVY, grand average of hydropathicity score.

### 3.2 Phylogenetic relationships and conserved motif analysis

**Figure 1:**
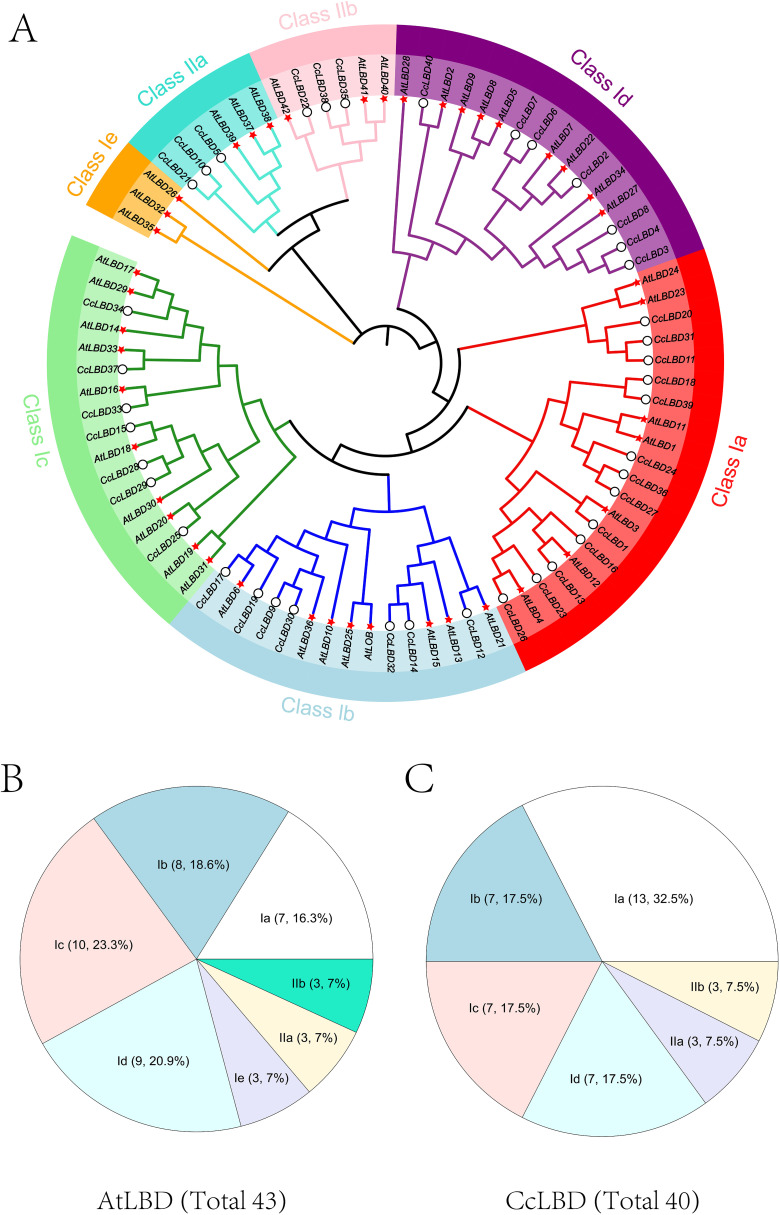
Phylogenetic relationships and subclass distribution of LBD proteins in *A. thaliana* (At) and *C. camphora* (Cc). **(A)** The circular cladogram delineates seven conserved clades (Class Ia, Ib, Ic, Id, Ie, IIa, IIb), with lineage-specific expansions highlighted by distinct colors. **(B)** Companion pie chart quantifies clade proportions: *A. thaliana* LBDs exhibit dominant Ic (23.3%) and Ib (18.6%) subclasses. **(C)** Companion pie chart quantifies clade proportions: *C. camphora* LBDs are enriched in Ia (32.5%) and IIb (17.5%) subclasses. Representative protein IDs (e.g., AtLBD39, CcLBD42) are annotated on terminal branches.

To delineate the phylogenetic relationships of LBD proteins, Neighbor-Joining analysis was performed using 43 A*. thaliana* and 40 C*. camphora* LBD homologs (**Figure 1**) (Shuai et al., 2002). The CcLBD family was phylogenetically stratified into Class I (subdivided into Ia:13, Ib:7, Ic:7, Id:7) and Class II (IIa:3, IIb:3), revealing lineage-specific expansion patterns. The absence of subclass Ie in *C. camphora* indicates evolutionary divergence from *A. thaliana*.

**Figure 2:**
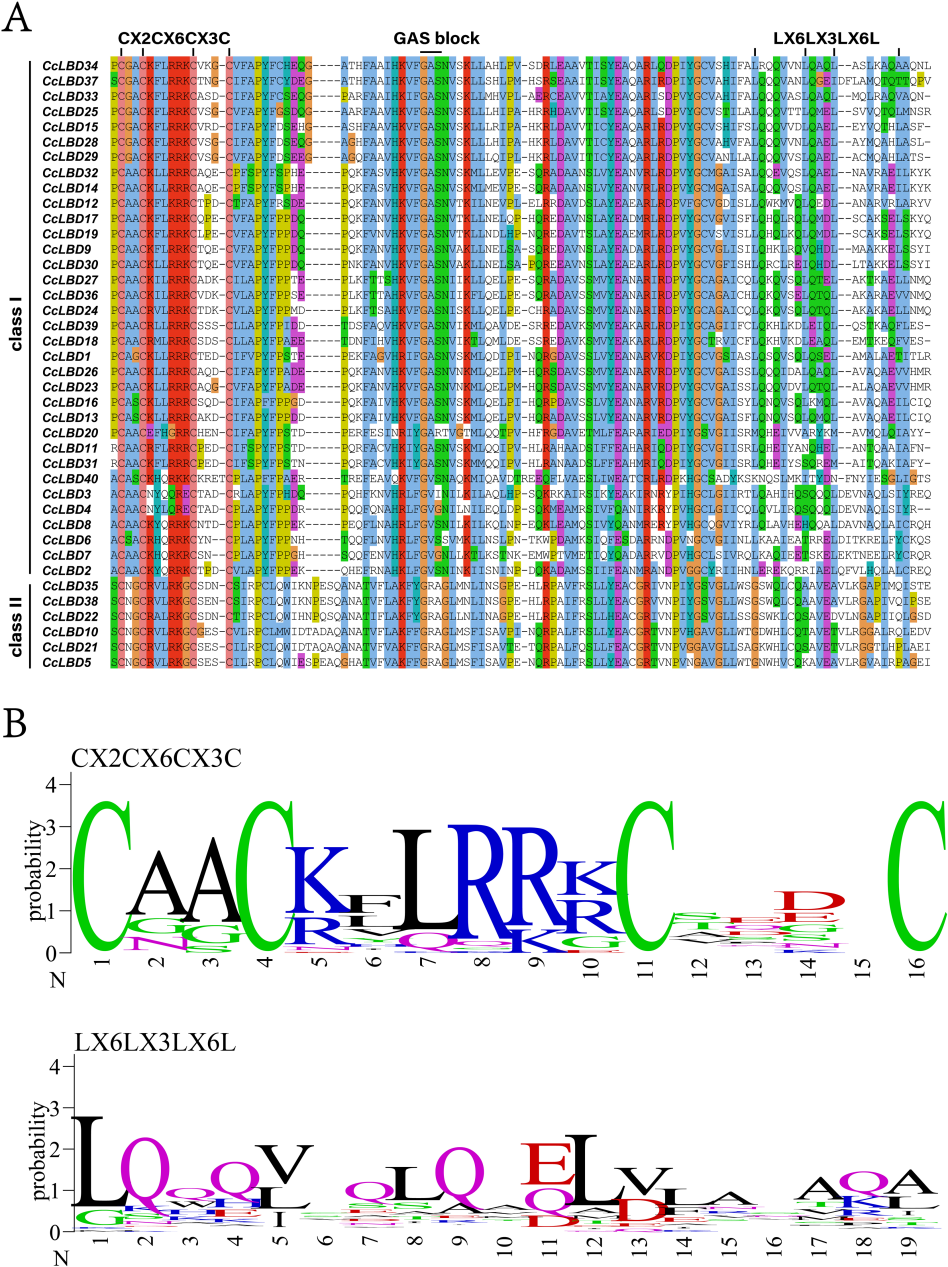
Conserved domain architecture of *C. camphora* LBD proteins. **(A)** Ubiquitous zinc finger-like CX2CX6CX3C domain across all 40 CcLBDs (color-coded), contrasting with Class I-specific leucine zipper-like LX6LX3LX6L motifs. **(B)** Domain sequence alignment (ClustalX 2.0) and corresponding motif logos (WebLogo-generated).

**Figure 3:**
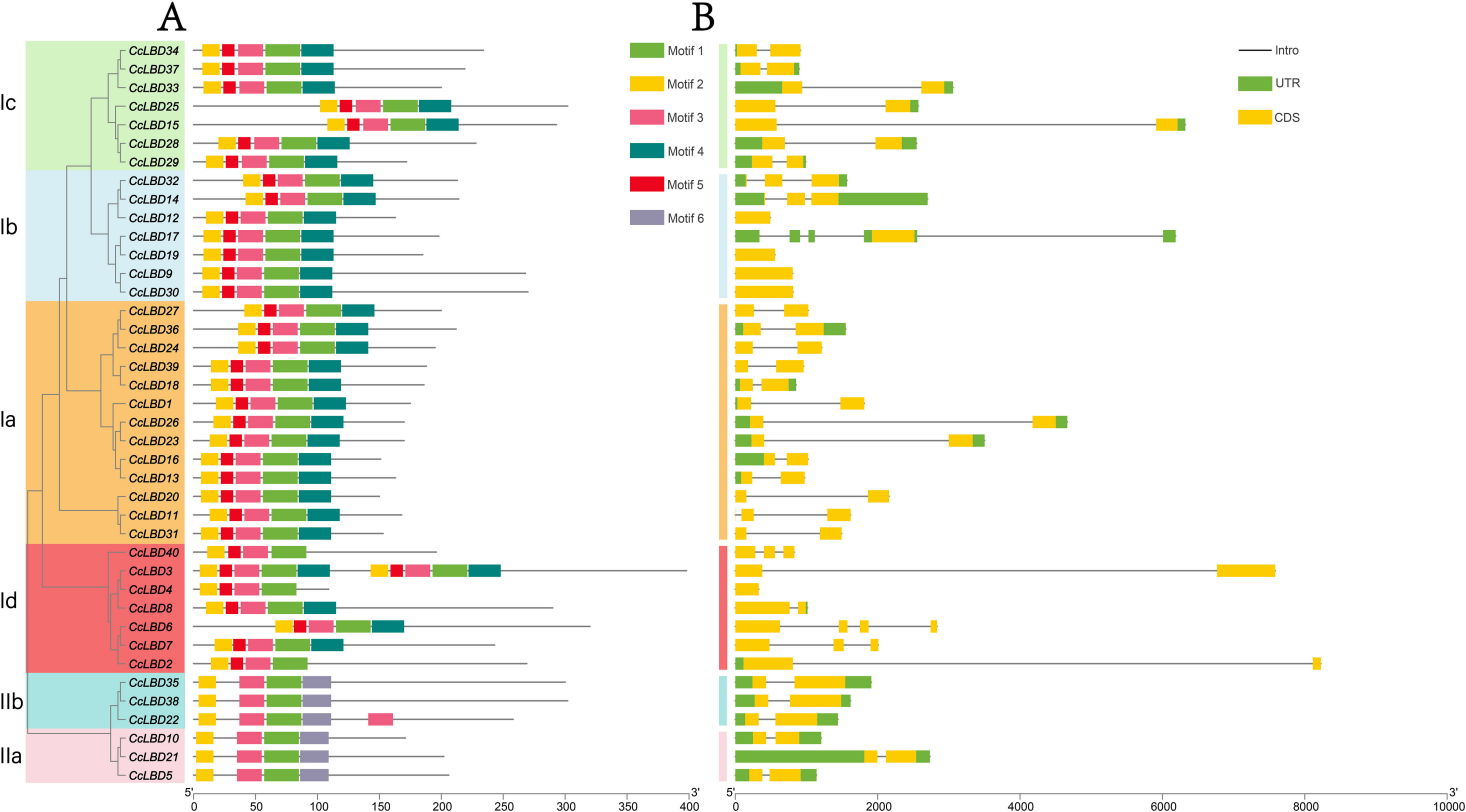
Conserved motifs and gene structures of CcLBDs. **(A)** Distribution of conserved motifs. The zinc finger-like ​CX2CX6CX3C​ domain is ubiquitous across all 40 CcLBDs, whereas the leucine zipper-like ​LX6LX3LX6L​ motif is restricted to Class I members. **(B)** Gene structure organization. Exon-intron architectures were analyzed via ClustalX2-based alignment and WebLogo-generated motif logos. Scale bars indicate gene length (bp) and protein sequence length (aa).

Conserved domain analysis delineated two signature architectures across CcLBD proteins: a 100-aa N-terminal LOB domain (**Figure 2A**) and a zinc finger-like motif (**Figure 2B**). Evolutionary conservation was evident as Class I members retained the angiosperm-typical leucine zipper domain, while complementary MEME analysis resolved six conserved motifs (**Figure 3A**). Notably, motifs 1–3 (LOB-associated) showed 97.5% conservation, whereas CcLBD3 uniquely harbored motifs 1–5, contrasting with the Class II-specific motif 6 (**Supplementary Figure S1**). Subclass specialization was observed, exemplified by motif six exclusivity to subclass II.

### 3.3 Gene structure and promoter elements

Gene structure investigation offers essential perspectives on the evolutionary trajectories of plant gene families (Li et al., 2017). Systematic alignment of *CcLBD* coding sequences with genomic DNA delineated the architectural organization of UTRs, exons, and introns (**Figure 3B**). Exon number variation was observed across the 40 *CcLBDs*, spanning 1–5 exons: 72.5% (29 genes) exhibited a two-exon configuration, contrasting with minority populations of single-exon (12.5%, five genes), three-exon (10%, four genes), and rare four-/five-exon (2.5%, one gene each) architectures. Subfamily-specific conservation of exon-intron structures was evident, displaying homologous exon length distributions and intron retention patterns. Mechanistically, intronic sequences mediated alternative splicing events and transcriptional fine-tuning, thereby expanding proteomic diversity through isoform generation (Greenwood and Kelsoe, 2003).

**Figure 4:**
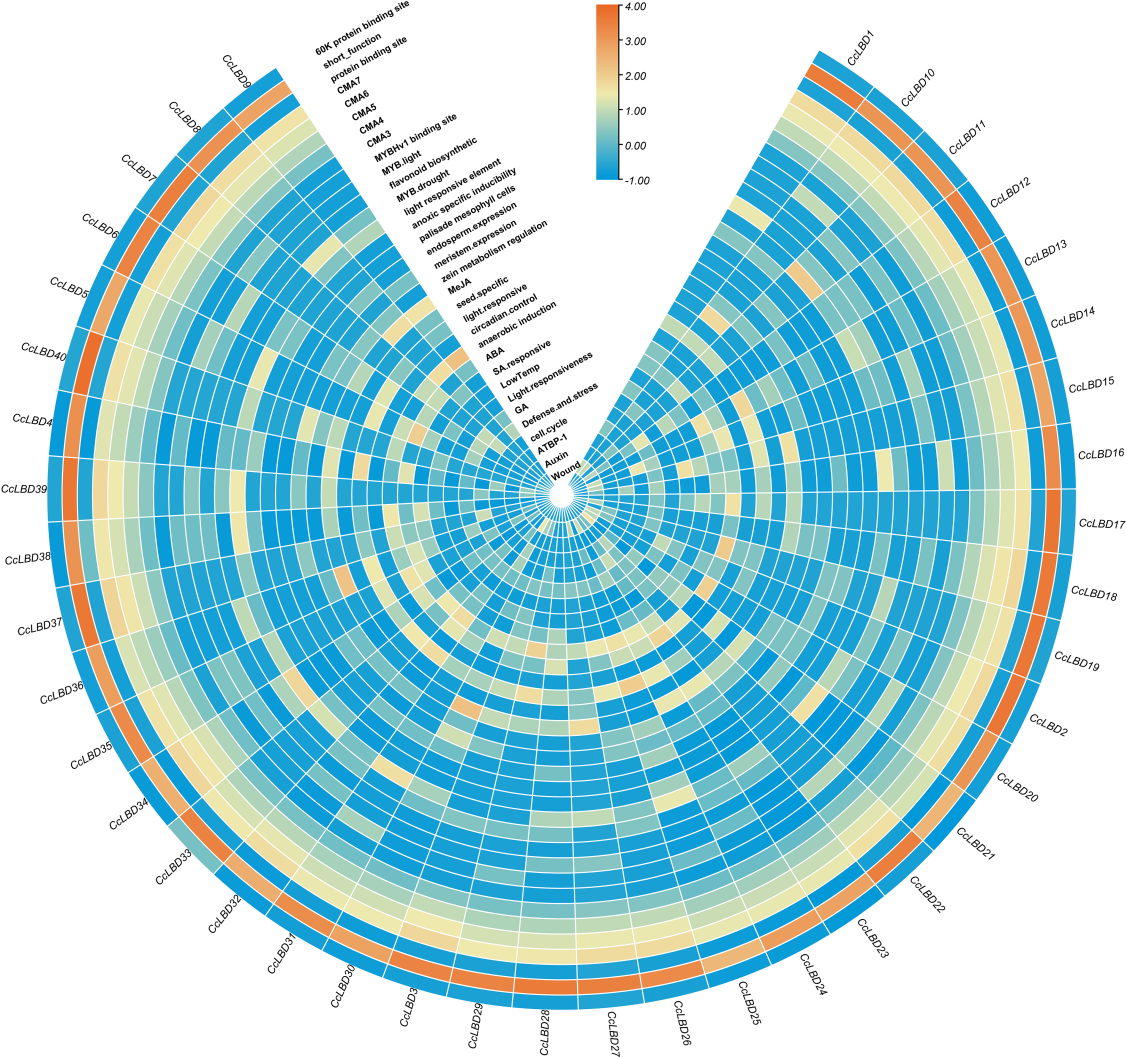
Cis-regulatory element profiling in *C. camphora LBD* promoters. The heatmap illustrates 33 functionally annotated cis-regulatory elements identified within 2,000-bp upstream promoter regions of 40 *CcLBD* genes. These elements include hormone-responsive motifs (e.g., ABRE, CGTCA, GARE, TCA, TGA) and stress-related elements involved in defense signaling, salt tolerance, and abiotic stress responses. Values represent log_2_-transformed counts of each element per gene, followed by row-wise Z-score normalization.

Promoter cis-element profiling of 2000-bp upstream regions from all *CcLBD* genes revealed 33 functional elements through PlantCare database annotation (**Figure 4**). Hormone-related elements encompassed ABRE (abscisic acid), CGTCA (MeJA), GARE (gibberellic acid), TCA (salicylic acid), and TGA (auxin) motifs. Stress-responsive elements included those associated with defense mechanisms, salt tolerance, and abiotic stress adaptation. These results indicate that *CcLBD* gene expression is modulated by cis-regulatory networks coordinating developmental processes and stress responses.

### 3.4 Genomic location and gene duplication events

**Figure 5:**
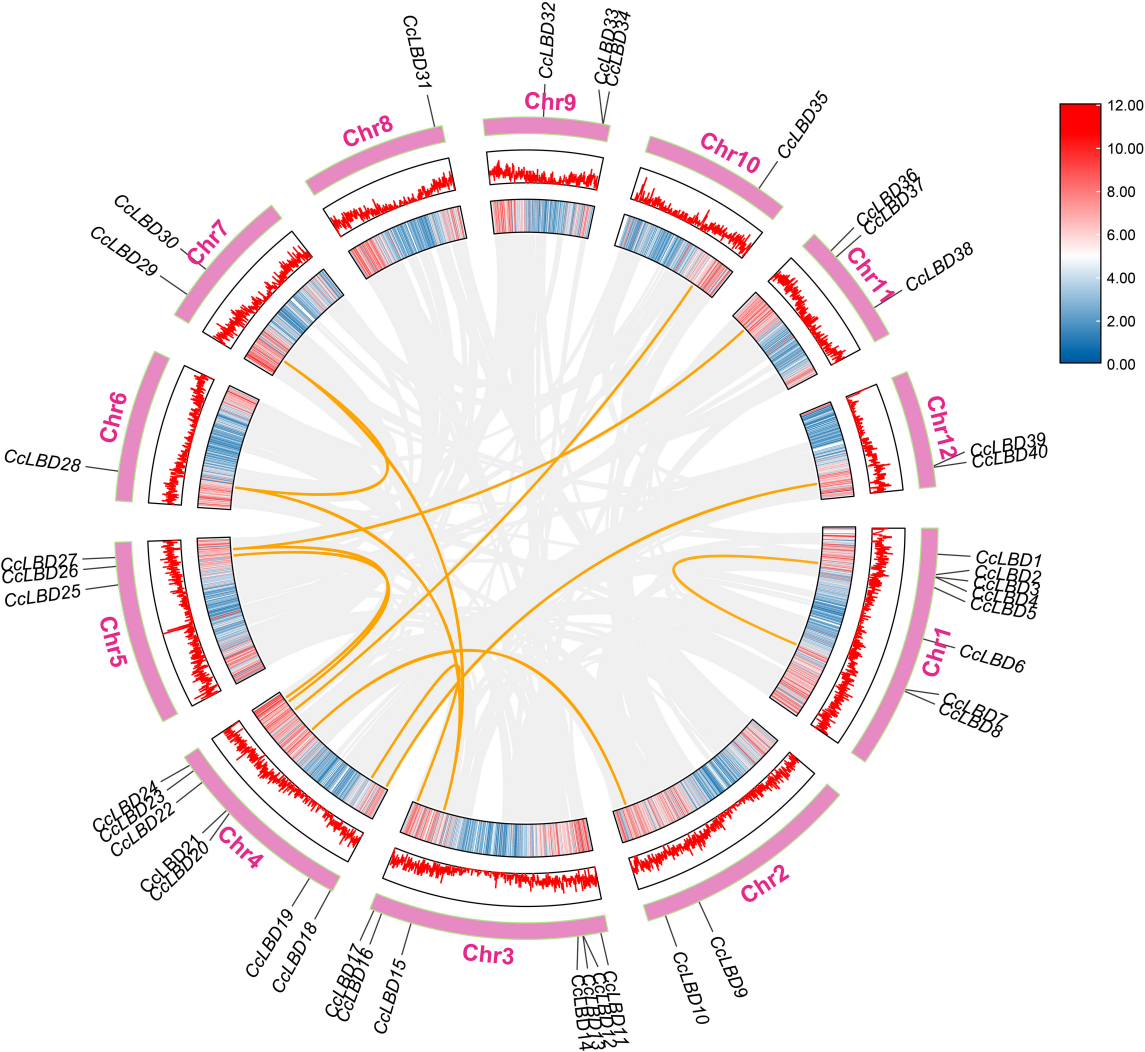
Chromosomal distribution and intraspecific collinearity of *C. camphora LBD* genes. *CcLBD* loci are annotated on chromosomes, with orange connectors marking tandem duplication events. The grey background lines represent syntenic relationships among *C. camphora* chromosomes, highlighting conserved genomic regions and gene collinearity across the genome.

The 40 *CcLBD* genes in the camphor tree are unevenly distributed across 12 chromosomes (**Figure 5**). Specifically, chromosomes 6, 8, and 10 each contain one *CcLBD* gene; chromosomes 2, 7, and 12 each harbor two genes; chromosomes 5, 9, and 11 each have three genes; chromosomes 3 and 4 each possess seven genes; and chromosome 1 harbors the highest number, with eight genes.

Evolutionary analysis revealed duplication events among *CcLBD* genes (**Figure 5**). Among the 40 *CcLBD* genes, 27.5% (11 genes) originated from duplication events. Chromosomal distribution analysis revealed that ​chromosomes 8 and 9​ lacked duplicated *CcLBDs*, whereas ​chromosome 4​ harbored the highest duplication frequency (six gene pairs).

Evolutionary analysis of *CcLBD* duplicates employed Ka/Ks substitution rate calculations to estimate divergence timing. The Ka/Ks ratio (nonsynonymous/synonymous substitution rates) for duplicated *CcLBD* gene pairs spanned 0.0139–0.3828, with all ratios < 1 (mean = 0.157), demonstrating strong purifying selection (**Supplementary Table S1**) (Zhang et al., 2006).

### 3.5 Differential expression profiles

**Figure 6:**
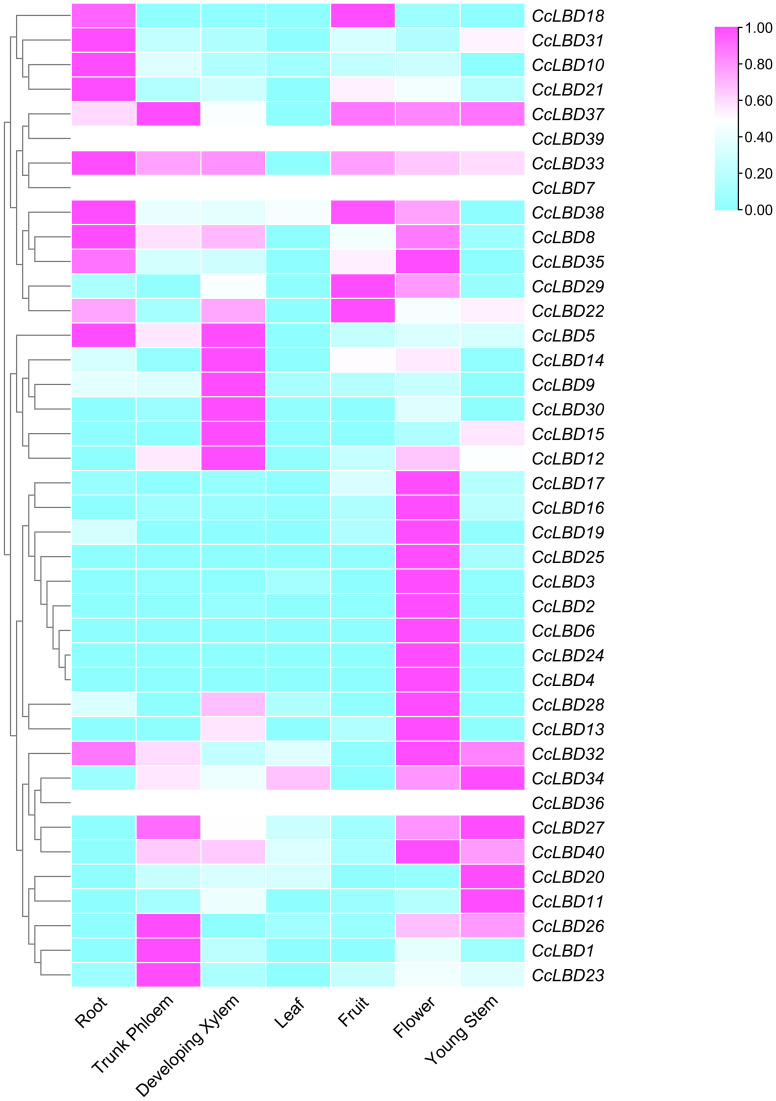
Transcriptional landscape of *CcLBD* genes in *C. camphora*. The heatmap displays log_10_(TPM+1) normalized expression values for 40 *CcLBDs* across seven tissues (root, trunk phloem, developing xylem, leaf, fruit, flower, young stem). Expression gradients are color-coded: purple (high) to blue (low).

Transcriptome profiling is critical for elucidating gene functions in plant growth and development (Tong et al., 2013). Here, RNA-Seq was employed to assess transcriptional dynamics of 40 *CcLBD* genes across seven organs: flowers, leaves, fruits, roots, young stems, developing xylem, and trunk phloem. Expression levels were normalized to ​Transcripts Per Million (TPM)​ and visualized via heatmap (**Figure 6**). Distinct tissue-specific patterns emerged: ​22 *CcLBDs*​ showed elevated expression in flowers, whereas ​12 genes​ were root-enriched, suggesting organ-specific regulatory roles in *C. camphora* development. Intriguingly, genes within the same phylogenetic clade exhibited divergent expression patterns across tissues, implying functional diversification of *CcLBD* paralogs. Transcriptome profiling revealed pronounced tissue-specific expression heterogeneity among *CcLBDs*, with ​22 genes​ preferentially expressed in floral tissues and ​12 genes​ showing root-specific activation (**Figure 6**), suggesting specialized roles in organ development.

**Figure 7:**
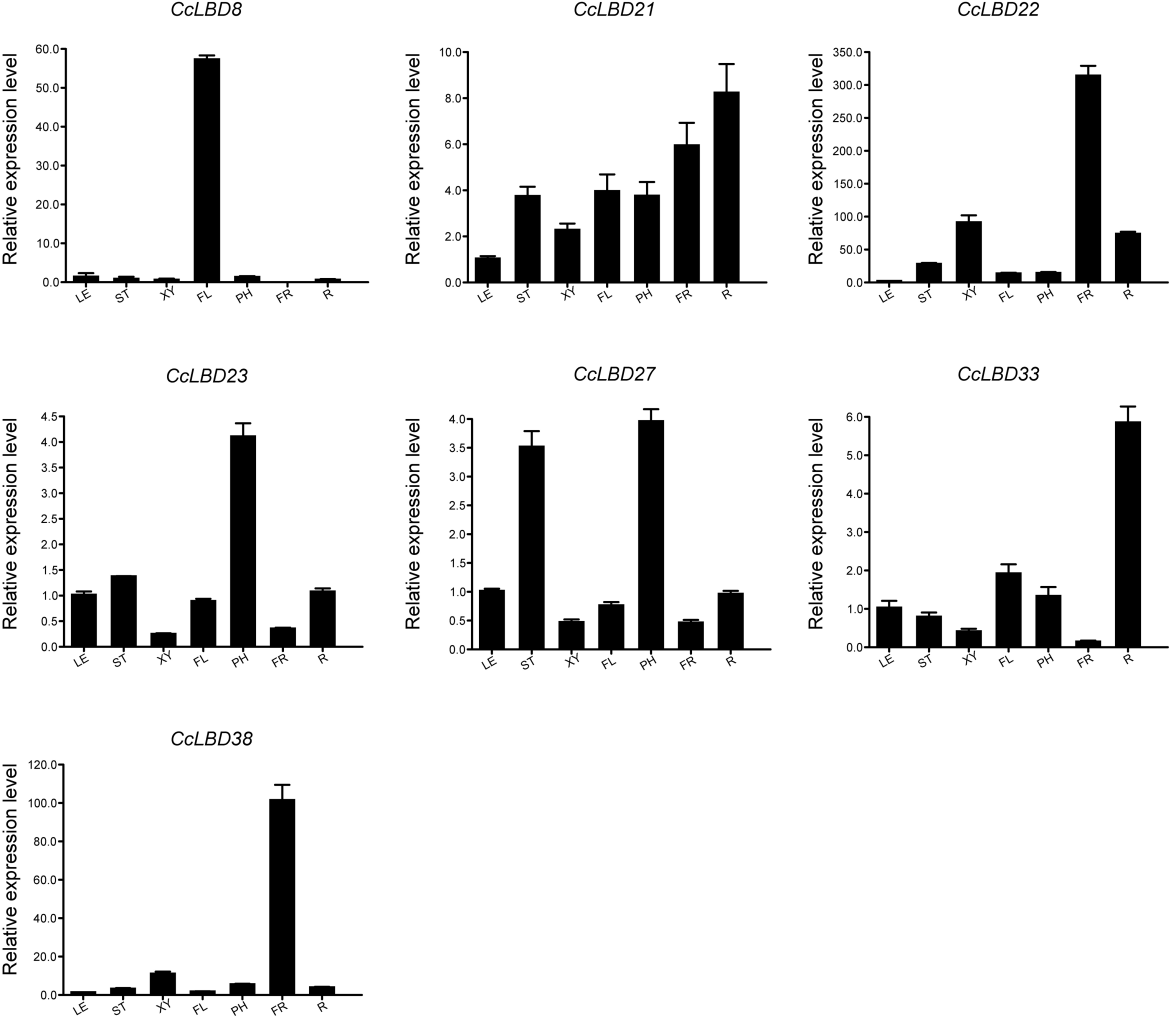
Tissue-specific expression profiles of seven *CcLBD* genes in *C. camphora*. Tissues analyzed: root, trunk phloem, developing xylem, leaf, fruit, flower, and young stem. Data represent mean ± SD from three biological replicates.

To validate the transcriptomic findings, we conducted qRT-PCR analysis on seven representative *CcLBD* genes across multiple tissues (**Figure 7**; **Supplementary Table S2**). The results corroborated the RNA-Seq data, revealing pronounced tissue-specific expression patterns and suggesting potential subfunctionalization within the *CcLBD* family. *CcLBD8* was exclusively expressed in flowers, indicating a specialized role in floral organ development. *CcLBD21* displayed broad expression, with the highest transcript abundance in roots, followed by fruits and trunk phloem, suggesting its involvement in root development and secondary tissue formation. *CcLBD22* and *CcLBD38* were both highly expressed in fruits, implying a possible function in fruit maturation. *CcLBD23* and *CcLBD27* exhibited predominant expression in trunk phloem, with *CcLBD27* also showing moderate expression in young stems, implying a coordinated role in phloem differentiation and early secondary vascular development. *CcLBD33* demonstrated root-specific expression, pointing to a potential function in root patterning. These findings not only validate the RNA-Seq results but also underscore the functional divergence among *CcLBD* members, with distinct expression profiles reflecting specialized roles in organ development and physiological regulation in *C. camphora*.

### 3.6 Regulation of *CcLBD5* by microRNAs in the camphor tree

**Figure 8:**
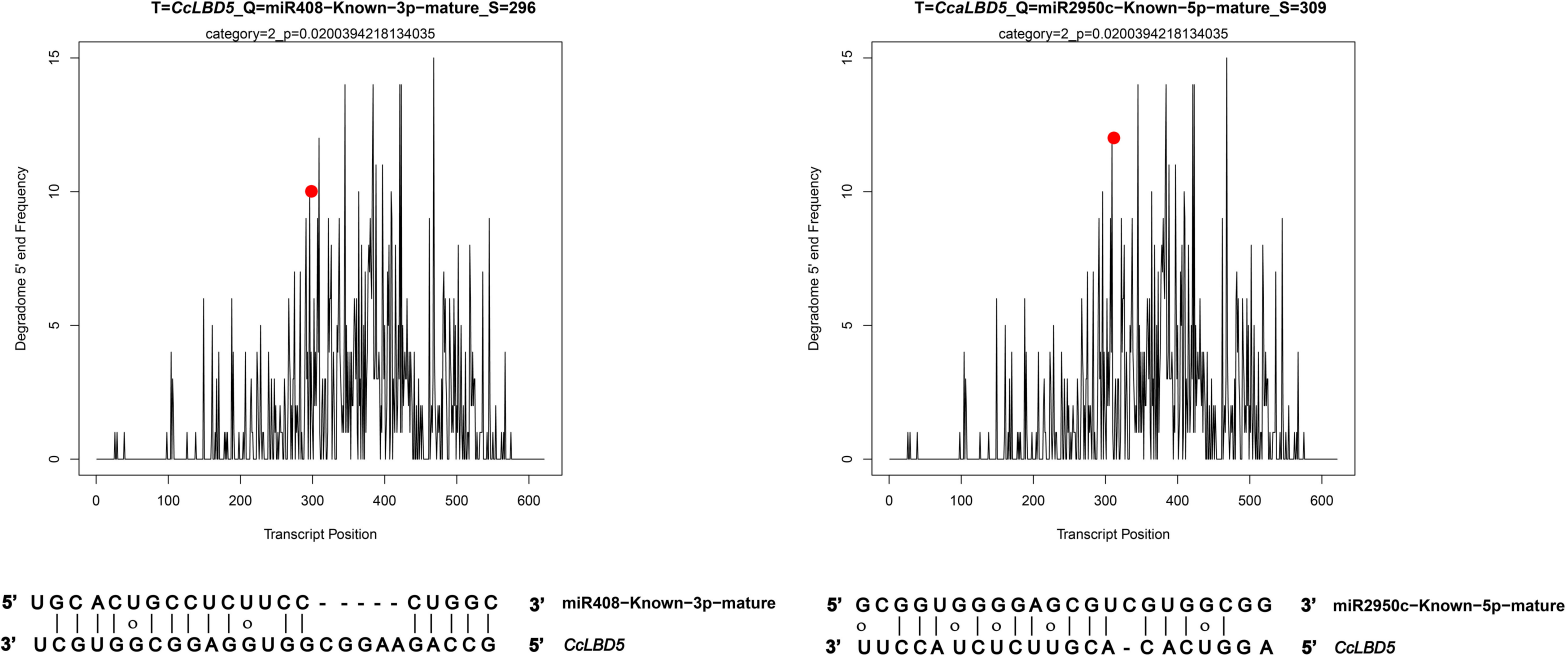
Degradome sequencing t-plots validate miR408 and miR2950c targeting *CcLBD5* at positions 296 and 309, respectively.

In this study, CleaveLand4 (version 4.5), a computational tool, was employed to categorize miRNA-mRNA interactions into three confidence levels (0, 1, and 2), facilitating a more accurate identification of potential regulatory miRNA-target pairs. Through degradome sequencing and subsequent computational analysis, two specific miRNA-*CcLBD* gene interactions were identified and characterized (**Figure 8**). Degradome analysis identified miR408 and miR2950c as post-transcriptional regulators of *CcLBD5*, with cleavage sites at positions 296 and 309, respectively. These findings demonstrate miRNAs’ regulatory impact on *CcLBD* gene networks, likely mediating *C. camphora*’s developmental plasticity and stress adaptation.

## 4 Discussion

The LBD transcription factor family regulates plant-specific developmental pathways and demonstrates evolutionary conservation across angiosperms, as evidenced in *A. thaliana* and *Oryza sativa* (Majer and Hochholdinger, 2011; Xu et al., 2016). Utilizing whole-genome annotation approaches, we systematically identified 40 *CcLBD* genes in the *C. camphora* genome, revealing conserved domain architectures characteristic of this plant-specific TF family. These proteins were categorized by a conserved C-terminal ​leucine zipper-like domain​ (LX6LX3LX6L), with ​Class I​ containing 85% of members and ​Class II​ comprising the remaining 15%. This distribution reveals strong evolutionary selection for Class I expansion, mirroring patterns observed in model systems like *A. thaliana* and *O. sativa* (Shuai et al., 2002; Yang et al., 2006).

While the core domains of LBD proteins remain evolutionarily conserved, their biological functions have undergone significant diversification across plant lineages. In annual species like *A. thaliana*, key LBD proteins (e.g., AtLBD18, AtLBD29, and AtLBD30) primarily regulate lateral root formation and tracheary element differentiation through auxin-mediated signaling pathways (Soyano et al., 2008; Pandey et al., 2018). Similarly, in species such as *S. lycopersicum* and *M. truncatula*, LBD homologs are involved in adventitious root formation and nodule development (Schiessl et al., 2019; Soyano et al., 2019; Omary et al., 2022). In contrast, in perennial woody species such as *Populus* and *Eucalyptus*, LBD proteins are predominantly associated with secondary vascular development and wood formation, particularly through the regulation of xylem and phloem differentiation (Yordanov et al., 2010; Lu et al., 2018; Yu et al., 2022). Our qRT-PCR analysis revealed that several *CcLBD* genes (*CcLBD21*, *CcLBD23*, and *CcLBD27*) exhibit high expression in trunk phloem and developing xylem, suggesting their involvement in secondary growth regulation. These findings are consistent with the established roles of LBD proteins in other woody species and support the hypothesis that LBD proteins have evolved specialized functions to govern long-term growth processes in perennial plants.

Structural analysis of the *CcLBD* genes revealed a relatively conserved gene organization, with members of each subgroup exhibiting comparable exon/intron configurations. This structural uniformity among the *CcLBD* genes is in line with previous findings in *A. thaliana*, *O. sativa*, and *Malus domestica* (Shuai et al., 2002; Yang et al., 2006; Wang et al., 2013), suggesting a conserved structural basis for the *LBD* family across different angiosperms. Conserved motif analysis revealed that all CcLBD subclasses share motifs 1-3, which collectively constitute the conserved LOB domain architecture. Subclass-specific motifs were observed, with motif six exclusively localized to Class II members, reflecting functional divergence within the LBD family. A highly conserved CX2CX6CX3C zinc finger-like motif, critical for DNA binding, further underscores the transcriptional regulatory capacity of these proteins.

The promoter regions of *CcLBD* genes exhibit significant enrichment of signal-responsive cis-elements, suggesting coordinated transcriptional regulation during environmental adaptation. In *Arabidopsis*, LBD15 enhances drought tolerance via ABA-mediated transcriptional activation of *ABSCISIC ACID INSENSITIVE4* (*ABI4*) (Guo et al., 2020). Similarly, ​*ZmLBD5*​ adjusts gibberellin and abscisic acid homeostasis to mediate drought adaptation in *Zea mays* (Feng et al., 2022). In root development, ​*AtLBD16*/*28*/*29*/*33*​ orchestrate lateral root formation via auxin signaling, a process dependent on *ARF7* and *ARF19*-mediated auxin response elements (Okushima et al., 2007; Lee et al., 2009). Additionally, methyl jasmonate (MeJA) regulates *LBD* genes in *Gossypium* species, underscoring the intricate crosstalk between phytohormones and transcriptional networks (Li et al., 2020). Genome-wide screening identified 41 cis-regulatory elements associated with phytohormone signaling pathways (ABA, MeJA, IAA) in *CcLBD* promoter regions. Notably, 15 ABA-responsive elements (ABREs) and 11 stress-related motifs were systematically annotated, with ABREs ubiquitously present across all *CcLBD* promoters. This comprehensive cis-element landscape suggests that *CcLBD* genes orchestrate *C. camphora*’s transcriptional reprogramming during abiotic stress adaptation.

MiRNAs are well-established as crucial post-transcriptional regulators of plant development, primarily through their targeting of transcription factors that, in turn, regulate downstream gene expression networks (Rong et al., 2024). Previous studies have demonstrated that miRNAs such as miR390 and miR408 are indirect modulators of LBD-mediated developmental pathways, acting via upstream regulators including members of the ARF and NAC transcription factor families (Guo et al., 2023; Kirolinko et al., 2024). In this study, we report the first evidence of potential direct miRNA–LBD interactions in *C. camphora*. Through integrated degradome sequencing and computational analyses, we identified miR408 and miR2950c as putative direct regulators of *CcLBD5*, suggesting a lineage-specific post-transcriptional regulatory mechanism in this woody species. While these predictions are supported by bioinformatic evidence and partial degradome data, further experimental validation is required to confirm these interactions. Future research should employ 5′ RACE (Rapid Amplification of cDNA Ends) to definitively characterize miRNA-guided cleavage events. Moreover, comprehensive screening of miRNA-binding sites across the entire *CcLBD* gene family could reveal the broader regulatory network mediated by miRNAs in perennial woody plants, providing crucial insights into their unique developmental regulation.

## 5 Conclusion

This study establishes the first genome-wide inventory of 40 *CcLBD* genes in *C. camphora*, phylogenetically classified into six distinct subclasses (Ia, Ib, Ic, Id, IIa, IIb). Comprehensive characterization integrated chromosomal distributions, exon-intron organizational patterns, conserved protein domain architectures, promoter cis-regulatory landscapes, and tissue-preferential expression dynamics. Expanding beyond transcriptional regulation, miRNA interaction networks were mapped to reveal post-transcriptional control nodes targeting *CcLBDs*, notably identifying miR408 and miR2950c as key post-transcriptional regulators. These collective insights advance the functional annotation of *LBD* genes in camphor trees and provide a mechanistic scaffold for probing their roles in lineage-specific developmental adaptations.

## Data Availability

The datasets presented in this study can be found in online repositories. The names of the repository/repositories and accession number(s) can be found in the article/**Supplementary Material**.
