# Supplementary figures and images for: Genome-wide identification, transcriptional profiling, and miRNA-binding site analysis of the LBD gene family in the camphor tree
Source: Front Plant Sci. 2025 Jun 19;16:1591736. doi: 10.3389/fpls.2025.1591736 (PMC12222220; doi:10.3389/fpls.2025.1591736)

Figure S1. The LOGO of six amino acid motifs in LBD proteins.

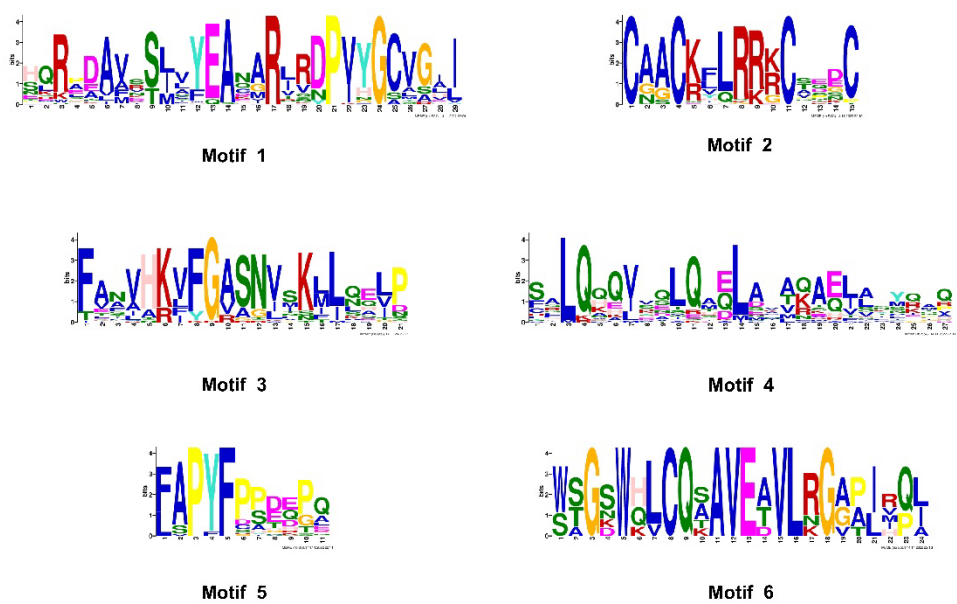

Supplement: Supplementary file 1 [file DataSheet1.pdf]
